# Mast Cells: A New Frontier for Cancer Immunotherapy

**DOI:** 10.3390/cells10061270

**Published:** 2021-05-21

**Authors:** Jake N. Lichterman, Sangeetha M. Reddy

**Affiliations:** 1 Division of Hematology/Oncology, Department of Internal Medicine, University of Texas Southwestern Medical Center, Dallas, TX 75390, USA; Jake.Lichterman@UTSouthwestern.edu; 2 Harold C. Simmons Comprehensive Cancer Center, University of Texas Southwestern Medical Center, Dallas, TX 75390, USA

**Keywords:** mast cell, cancer, immunotherapy, microenvironment, cancer immunology, c-KIT, TLR, toll-like receptors

## Abstract

Mast cells are unique tissue-resident immune cells of the myeloid lineage that have long been implicated in the pathogenesis of allergic and autoimmune disorders. More recently, mast cells have been recognized as key orchestrators of anti-tumor immunity, modulators of the cancer stroma, and have also been implicated in cancer cell intrinsic properties. As such, mast cells are an underrecognized but very promising target for cancer immunotherapy. In this review, we discuss the role of mast cells in shaping cancer and its microenvironment, the interaction between mast cells and cancer therapies, and strategies to target mast cells to improve cancer outcomes. Specifically, we address (1) decreasing cell numbers through c-KIT inhibition, (2) modulating mast cell activation and phenotype (through mast cell stabilizers, FcεR1 signaling pathway activators/inhibitors, antibodies targeting inhibitory receptors and ligands, toll like receptor agonists), and (3) altering secreted mast cell mediators and their downstream effects. Finally, we discuss the importance of translational research using patient samples to advance the field of mast cell targeting to optimally improve patient outcomes. As we aim to expand the successes of existing cancer immunotherapies, focused clinical and translational studies targeting mast cells in different cancer contexts are now warranted.

## 1. Introduction

Cancer immunotherapy—in particular, immune checkpoint blockade (ICB)—has transformed oncology care in the last decade and significantly improved survival in a wide range of metastatic tumors and more recently has improved outcomes in earlier stage disease. Based on significant treatment benefit, ICB treatments are FDA approved either as monotherapy or in combination with other cancer therapies in melanoma, breast cancer, renal cell carcinoma, head and neck squamous cell carcinoma, and lung cancer, among others [1,2,3,4,5]. Unfortunately, while responders derive significant benefit from therapy, many patients do not have treatment responses. While currently, programmed cell death protein 1 (PD-1), programmed cell death ligand 1 (PD-L1), and cytotoxic T-lymphocyte-associated protein 4 (CTLA-4) immune checkpoint blockade are clinically approved, other immune checkpoint targets are under clinical investigation. Initial investigation of cancer immunotherapies has focused on T-cell targeted therapies such as ICB because T-cells are considered primary effector cells of anti-tumor immunity and induce particularly long-lasting memory responses. There is also increasing research into targeting upstream determinants of T-cell activation such as antigen-presenting dendritic cells that initiate the cancer immunity cycle with T cells [6]. Recently, there is a growing awareness of the importance of other immune cells in shaping cancer outcomes and anti-tumor immunity, including mast cells, which we recently described to be associated with chemoresistance in breast cancer [7].

Mast cells are tissue-resident myeloid cells present throughout the connective tissues in our body that contain coarse granules with potent inflammatory mediators such as histamine. While they are traditionally associated with allergy and inflammation, mast cells are now recognized to critically shape tumor cell and tumor microenvironment behavior. In this review, we examine the role of mast cells as context dependent and highly plastic mediators of anti-tumor immunity and discuss strategies to therapeutically manipulate them to elicit durable immune responses as monotherapy or in combination with T-cell-targeted approaches such as immune checkpoint blockade. In addition to reviewing existing pre-clinical and clinical data in oncology, we address mast cell targeting modalities used in other fields such as allergic diseases. While there is extensive pre-clinical and some translational research regarding the relationship between mast cells and cancer pathogenesis or outcomes, targeting mast cells as a therapeutic strategy in human cancer patients is in its relative infancy. We therefore address the importance of a translational research approach that utilizes samples from cancer patients to identify optimal mast-cell-targeting therapies and determine future rational therapeutic combinations.

## 2. Mast Cell Background

Mast cells are derived from CD34+ bone marrow myeloid precursors that circulate in the blood and migrate to peripheral tissues where they develop and differentiate into mature mast cells under the pressure of tissue specific chemokines and cytokines (such as stem cell factor and IL-4), extracellular matrix proteins, and adhesion molecules. Mast cells are strategically located throughout the body near blood vessels, lymphatics, and mucosal surfaces such as the skin and gastrointestinal tract, where they interface with the external environment. Their location allows them to mediate systemic responses to local stimuli and orchestrate important aspects of both innate and adaptive immunity as well as other physiologic processes.

Mast cells have numerous stimulatory and inhibitory ligands that lead to integration of incoming signals and secretion of various stored mediators in secretory granules as well as newly synthesized mediators. Pre-formed secretory granules contain proteases such as tryptase and chymase, histamines, heparin, lysosomal enzymes, and inflammatory cytokines such as TNF-α that are implicated in severe allergic diseases such as urticaria and anaphylaxis. Mast cells exhibit substantial heterogeneity in their granule content, with one subtype classification distinguishing based on whether they contain tryptase without chymase (MC_T_), chymase without tryptase (MC_C_), or both (MC_TC_) [8,9]. Murine studies suggest that mast cell phenotypes are versatile and can change based on the surrounding microenvironment including cytokine exposure as well as the stage of mast cell development [8,10,11,12,13,14]. Two types of degranulation have been described: anaphylactic degranulation in which entire granule contents are rapidly released to the extracellular environment, or piecemeal degranulation in which only a portion of the contents of pre-formed granules are released in a more graded and specific manner [9,15]. Both of these have been described in humans as well as other species. In addition to or separately from degranulation, mast cell activation can result in the release of various pre-formed but also largely de novo synthesized growth factors, eicosanoids including prostaglandins, leukotrienes, chemokines such as CXCL10, and cytokines [8,16,17]. Cytokines shown to be secreted by mast cells include inflammatory cytokines such as TNF, IL-6, and IL-1, but also anti-inflammatory cytokines such as IL-10 and TGF-ß; mast-cell-derived growth factors, cytokines, and chemokines are reviewed more thoroughly elsewhere [16].

The most well-studied mechanism through which mast cell degranulation occurs is antigen-specific immunoglobulin E (IgE) cross-linking of the high-affinity IgE-bearing surface receptor FcεRI following exposure to a cognate antigen leading to rapid mast cell degranulation [17]. Mast cells can also be activated via alternative mechanisms such as by damage-associated and pathogen-associated molecular patterns through toll-like receptors, complement proteins, cytokines, and other stimuli. Substantial heterogeneity among mast cells in the expression of different surface receptors such as complement receptors has been demonstrated with resulting functional consequences, though our understanding of the mechanism for this differential expression is limited [8,18,19,20].

**Figure 1:**
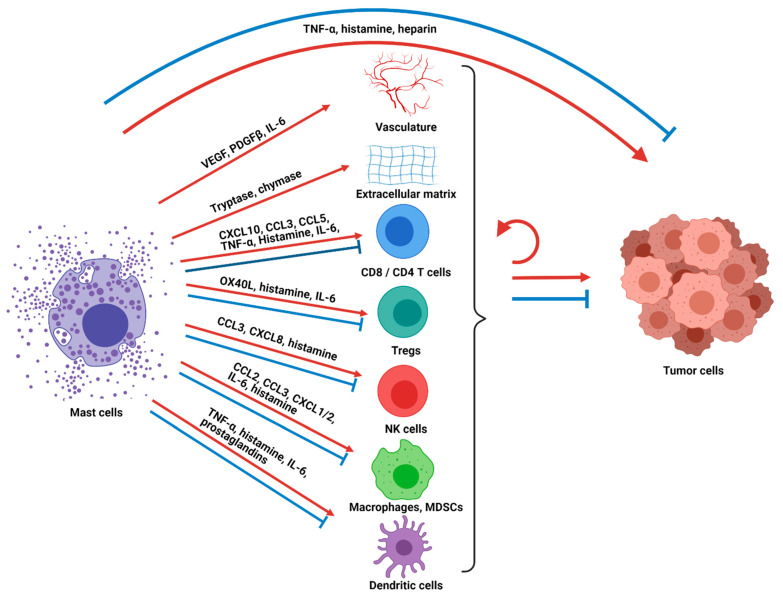
Mast cells are orchestrators of anti-tumor immunity and tumor control. Mast cells directly impact tumor cells as well as immune and non-immune components of the tumor microenvironment through chemokine secretion and release of other mediators, leading to varied cancer-promoting or cancer-suppressive properties. These components of the tumor microenvironment can further interact with each other and/or the tumor cells directly, with the net cumulative signals determining the impact on tumor control. TNF-α, tumor necrosis factor alpha; VEGF, vascular endothelial growth factor; PDGF-β, platelet derived growth factor beta; IL-6, interleukin 6; CXCL10, C-X-C motif chemokine ligand 10; CCL3, chemokine ligand 3; CCL5, chemokine ligand 5; OX40L, OX40 ligand; CXCL8, C-X-C motif chemokine ligand 8; CCL2, chemokine ligand 2; CXCL1/2, chemokine ligand 1 or 2; Tregs, regulatory T cells; NK cells, natural killer cells; MDSCs, myeloid-derived suppressor cells.

The net results of mast cell activation, degranulation, and/or secretion of inflammatory mediators include activation or attraction of other immune, stromal, neuronal, and epithelial cells which lead to changes in the local tissue microenvironment such as vasodilation and angiogenesis and also activation of systemic immune responses (Figure 1). Mast cell activation and/or degranulation can happen in the classical rapid manner leading to a massive release of inflammatory mediators and dramatic clinical presentations such as anaphylaxis and angioedema. However, these processes can also occur gradually with the slow release of specific mediators leading to chronic inflammatory and local tissue changes. This latter form of mast cell activation is particularly relevant in cancer where mast cells have been seen to function as central regulators of tissue remodeling and as sentinel immune cells that coordinate innate and adaptive immune responses [21].

## 3. Mast Cells in Cancer and Anti-Tumor Immunity

Tumor-associated mast cells have been observed in the solid tumor microenvironment of numerous cancers and have intriguingly been found to be a favorable prognostic factor in some cancers, such as esophageal adenocarcinoma, ovarian cancer, and diffuse large B-cell lymphoma [22,23,24], while they are associated with a poor or mixed prognosis in other cancers, such as gastric cancer, lung cancer, melanoma, and breast cancer [7,25,26,27,28,29]. There is also growing awareness that mast cells are a biomarker and important determinant of cancer treatment responses. We recently demonstrated that higher pre-treatment mast cell infiltration is significantly associated with poor responses to pre-surgical chemotherapy in an aggressive form of localized breast cancer [7], and recent data show that higher mast cell tumor infiltration predicts poor responses to anti-PD-1 ICB in melanoma [30]. Mast cells can accumulate in cancers due to various growth factors and chemokines, including stem cell factor (SCF), vascular endothelial growth factor (VEGF), CCL2, IL-8, complements, and PGE2 [21,31]. Due to the multifaceted nature of mast cells and their immunomodulatory effects upon activation or degranulation, mast cells have been found to be both pro- and anti-tumorigenic (Figure 1). As discussed above, there is substantial variation in mast cell phenotype and function and the extent to which they produce and release mediators based on anatomic location, stage of mast cell development, and exposure to environmental inflammatory mediators. Additionally, the downstream effects of secreted mediators are also heterogeneous based on the specific constitution of the surrounding environment and target cells. Therefore, the ability of mast cells to promote or impede tumorigenesis has been shown to be dependent on tumor type, cancer stage, the activation status of the mast cells, the location of the mast cells within the tumor microenvironment, and the net balance of pro- and anti-tumorigenic effects on the tumor cells [15]. For example, in a study of surgically resected non-small cell lung cancer patients, intratumoral mast cells but not stromal mast cells were associated with a favorable prognosis [32,33]. Similarly, analysis of prostate cancer samples shows that a higher number of mast cells in the tumor compartment had longer cancer-specific survival, whereas the inverse was seen with mast cell infiltration in the non-malignant stromal compartment [23].

Mast cells have been shown to directly impact the tumor cells as well as the surrounding tumor-associated stroma to alter tumor pathogenesis through multiple mechanisms (Figure 1). For example, mast cells can release large quantities of tumor necrosis factor alpha (TNF-α), which leads to direct tumor cell cytotoxicity [34], while in other contexts TNF-α promotes tumor growth [35]. Histamine is another secreted mast cell factor that has varied downstream effects depending on its surrounding context and which of its receptors (H_1_R, H_2_R, H_3_R, and H_4_R) are stimulated. Direct anti-proliferative as well as tumor-promoting effects have been observed on cancer cells [36,37,38]. Mast cells also release proteases such as tryptase and chymase that can activate matrix metalloproteinases that degrade the extracellular matrix and tissues around the tumor, allowing for tumor growth, angiogenesis and metastasis [39]. In addition, mast cells release VEGF, platelet-derived growth factor-β (PDGF-β) and IL-6 that promote angiogenesis, allowing for enhanced blood vessel formation, cellular proliferation and tumor growth [40,41]. Mast-cells also secrete IL-1, a pro-inflammatory cytokine that has been linked to tumorigenesis, tumor progression, and excessive inflammatory reactions [42,43]. Other mast cell mediators, such as heparin, prostaglandins, and other cytokines, also impact the non-immune aspects of the tumor and its environment.

A critical role for mast cells in modulating tumor progression is their role as a sentinel immune cell that releases chemokines, cytokines, and other factors that recruit other immune cells to the tumor microenvironment and alter their function. Located close to the vasculature, mast cells can translate local cues to systemic tumor modulating immune responses and also can be among the first cells to respond to systemic signals and be recruited themselves to sites of inflammation. Mast cells release chemokines such as CXCL10, CLL3, and CCL5 that recruit CD8 T cells and CD4 T cells to the tumor. After recruitment, they can modulate T-cell activity further [44,45,46,47,48,49,50,51,52] through TNF-α secretion that can enhance activation, or upregulate PD-L1 that can inhibit CD8 T cells. Mast-cell-secreted histamine can favor specific T helper subtypes or T regulatory responses depending on which receptor is stimulated. Secreted IL-6 can preferentially inhibit suppressive T regulatory cells, and direct OX40–OX40 ligand contact-dependent interactions can inhibit T regulatory cell functions relative to T effector cells. Activated mast cells have also been shown to upregulate MHC-II and costimulatory molecules to function as local antigen-presenting cells to T cells [44,53]. While several of these T-cell-modulating functions can promote anti-tumor immunity, other studies have shown that mast cell infiltration is associated with reduced IFN-γ producing CD8 T cells [54]. NK cells are another lymphoid cell subtype that can be recruited to the tumor microenvironment through mast-cell-secreted chemokines such as CCL3 and CXCL8 [21] and are activated in a contact-dependent manner by mast cells to secrete IFN-γ. Histamine secreted by mast cells has been shown to mediate changes to monocytes which in turn reduce immunosuppressive signals to NK cells [55,56,57].

Mast cells can also recruit and alter the function of myeloid cells such as tumor-associated macrophages, myeloid-derived suppressor cells, neutrophils and dendritic cells. Activated mast cells recruit tumor-associated macrophages by CSF2, CCL3, and IL-6 secretion, resulting in increased tumor growth, as shown in a gastric cancer murine model [58]. Myeloid-derived suppressor cells are recruited through CCL2 [59] and possibly CXCL1 and CXCL2 (which recruit neutrophils) to the tumor [60], where their suppressive activity is enhanced by direct contact with mast cells [61] or alternatively matured to less suppressive differentiated cells through histamine [62]. Dendritic cells are a critical myeloid cell subset that is considered the most potent antigen-presenting cell and stimulates antigen-specific T-cell immunity. However, depending on local cues, dendritic cells can stimulate anti-tumor phenotypes or can instead stimulate T regulatory cells and promote tolerance. Mast cells have been shown to promote dendritic cell migration to lymph nodes through TNF-α, histamine, and IL-6 and promote anti-tumor T-cell phenotypes through histamine. Alternatively, mast cells can promote immune tolerance through prostaglandins [63,64,65,66,67,68]. Mast cell degranulation has importantly been shown to counter T-cell tolerance [69]. Mast cells also secrete TGF-β and IL-10, which have suppressive effects on multiple immune cell subsets [70,71]. Mast-cell-derived TGF-β and IL-10 promote the development of IL-10-secreting T regulatory cells, downregulate costimulatory molecules on dendritic cells, decrease pro-inflammatory cytokines by macrophages, ultimately reduce antigen-specific T-cell responses, enhance fibrosis, and even regulate mast cells in an autocrine and paracrine manner [18,67,68].

The net balance of mast-cell-induced anti-tumor and tumor-promoting signals on the tumor, stroma, and immune microenvironment determines how tumor-associated mast cells impact final tumor growth. Tumor-directed cancer therapies such as chemotherapy, targeted therapies, radiation therapy, and immunotherapies further contribute to the tumor context that determines this balance. Appropriately understanding how these factors interact will therefore be important to studying relevant mast cell biology and how to optimally target mast cells as a therapeutic option to improve cancer outcomes.

## 4. Targeting Mast Cells for Cancer Therapy

**Figure 2:**
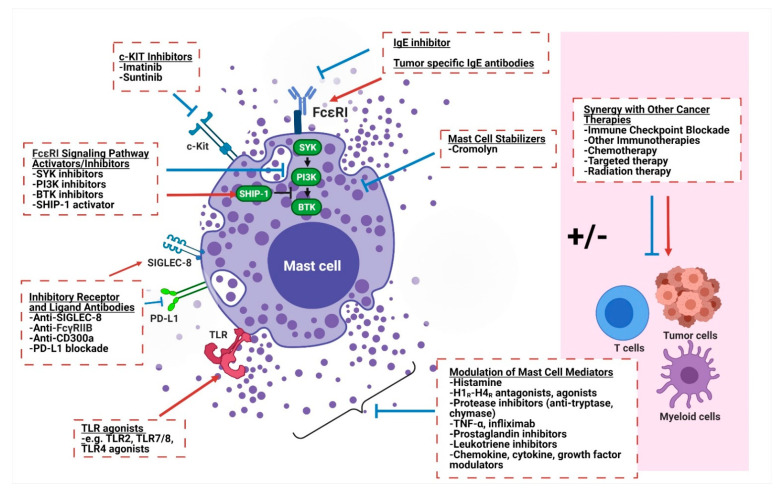
Therapeutic strategies to target mast cells for cancer immunotherapy. Mast cells can be therapeutically targeted by (1) decreasing cell numbers through c-KIT inhibition, (2) modulating mast cell activation and phenotype (through mast cell stabilizers, FcεR1 signaling pathway activators/inhibitors, antibodies targeting inhibitory receptors and ligands, TLR agonists), and (3) altering secreted mast cell mediators and their downstream effects. c-KIT, tyrosine protein kinase KIT or CD117; SYK, spleen tyrosine kinase; PI3K, phosphoinositide 3-kinase; BTK, Bruton’s tyrosine kinase; SHIP-1, Src homology 2 domain containing inositol polyphosphate 5-phosphatase 1; SIGLEC-8, sialic acid-binding Ig-like lectin 8; FcγRIIB, Fc gamma receptor IIB; PD-L1, programmed death ligand 1; TLR, toll-like receptor; TNF-α, tumor necrosis factor alpha; H1_R_-H4_R,_ histamine 1–4 receptor; FcεR1, high-affinity IgE receptor or Fc epsilon receptor 1; IgE, immunoglobulin E.

Based on the growing literature supporting the key role of mast cells in both promoting and decreasing tumor growth across cancers, they are a highly attractive therapeutic target for a variety of different malignancies. Therapeutics aimed at targeting mast cells in cancer have generally taken three approaches: (1) reducing mast cell numbers, (2) modulating mast cell activation and phenotype, and (3) altering secreted mast cell mediators and their downstream effects (Figure 2).

### 4.1. Reducing Mast Cell Numbers

Modulation of mast cell numbers has been a successful therapeutic strategy in disorders such as systemic mastocytosis in which mast cell numbers are pathologically increased. Mast cell numbers can be reduced by preventing terminal mast cell differentiation from myeloid precursor cells, decreasing growth factors needed for survival, or reducing recruitment of mast cells to the tumor. SCF is a cytokine that binds to the c-KIT receptor and is important for hematopoiesis and the regulation of hematopoietic stem cells, but later in hematopoietic development it is especially important for mast cell differentiation, survival, proliferation, and recruitment, as mast cells are one of the only terminally differentiated immune cells that express c-KIT [72]. Attempts at reducing or enhancing mast cell numbers through targeting c-KIT in a variety of solid tumors have been made using the tyrosine kinase inhibitor imatinib. Imatinib is a FDA-approved treatment for gastrointestinal stromal tumors which are driven by c-KIT mutations in >80% of cases, chronic myelogenous leukemia where it targets the aberrant BCR-ABL kinase, and in select metastatic melanoma tumors with activating c-KIT mutations [73,74,75]. Additional c-KIT-targeting therapies used in clinical practice include nilotinib, dasatinib, sunitinib, midostaurin, ibrutinib and masitinib [76]. Notably, mast cells are not the direct or intended targets of these drugs in the malignancies for which they are clinically used (other than systemic mastocytosis), and additionally these drugs are not specific to c-KIT but inhibit other receptors such as ABL kinase, Src kinase, and PDGFR-α and PDGFR-β. c-KIT-specific monoclonal antibodies such as CDX-1058 and CDX-0159 are in clinical development in inflammatory disease as well as in c-KIT-positive solid tumors (NCT02642016) and, unlike the tyrosine kinase inhibitors, are more specific to the intended target. In c-KIT-driven tumors, translational studies support the immunomodulatory role of c-KIT inhibition. Rusakiewicz et al. showed that imatinib treatment in gastrointestinal stromal tumors leads to a decrease in major histocompatibility class I molecules on tumor cells along with an increase in NK cell infiltration, which was associated with improved progression-free survival [77]. Unfortunately, mast cells were not assessed in this analysis, and it appears the immune effects are related to tumor-specific effects.

In cancers (not driven by c-KIT or Abl mutations), reducing mast cell numbers through c-KIT-targeting therapy alone has not yet proven to be a successful cancer strategy clinically or pre-clinically. For example, in the transgenic TRAMP mouse model of prostate cancer, while imatinib administration did decrease the development of well-differentiated prostate adenocarcinoma, it unexpectedly increased the incidence of an aggressive neuroendocrine phenotype of prostate cancer [78,79], raising a significant clinical concern that prevented translation to human studies. This discrepancy was felt to be due to defective signaling in the neuroendocrine tumors downstream of c-Kit. In the 4T1 mouse model of triple negative breast cancer, the administration of imatinib led to an increase rather than decrease in tumor mass as well as an increase in peri-tumoral blood clotting [80]. The authors concluded that this tumor growth was related to reduced mast cells and associated heparin secretion that would normally inhibit clotting and promote tumor control. Notably, the mast cell stabilizer sodium cromolyn also led to enhanced blood clotting and intratumoral hypoxia in this tumor model [80].

In contrast to the limited success with monotherapy studies, recent studies focusing on the immunomodulatory impact of mast cells suggest that depleting mast cells may synergize with other immunotherapeutics to most effectively control tumor growth. Using a humanized mouse model of melanoma, Somasundaram et al. identified that after PD-1 blockade, mast cells co-localize with T regulatory cells in regions of the tumor with reduced Granzyme B+ CD8+ immune cells and decreased HLA-class I expression, indicating a potential mechanism of resistance to PD-1 blockade [30]. While PD-1 blockade alone led to partial tumor control, complete regression of melanoma tumors was seen after depleting mast cells with sunitinib or imatinib. These mice further rejected rechallenged tumors indicating long-term memory T-cell responses. Notably, mast cell depletion alone in the absence of PD-1 blockade was insufficient at controlling tumor growth, underscoring the importance of the synergy between these two immunomodulatory approaches. Cedirinib, another tyrosine kinase inhibitor that instead targets VEGF and PDGF receptors, also did not demonstrate complete regression when combined with PD-1 blockade.

Mast cell infiltration could also be reduced by targeting chemoattractants in the tumor tissue that recruit mast cells to the tumor microenvironment. While SCF is an important mast cell chemokine, many of the other chemoattracts such as CCL2 and VEGF that attract mast cells have also been shown to attract other immune subtypes; it is also not clear which are most relevant to mast cells in humans [81]. Until we can clarify the importance of these chemokines in human cancers and their impact on mast cells, at this time these are suboptimal mast-cell-targeting therapies in cancer.

### 4.2. Modulating Mast Cell Activation and Phenotype

#### 4.2.1. Stabilizing Mast Cell Degranulation

Another therapeutic strategy for targeting mast cells under investigation is the prevention or abrogation of mast cell activation. Mast cell stabilizing agents that prevent degranulation are commonly used in allergic diseases, such as cromolyn sodium, and have been investigated in different preclinical models of solid tumors. In a xenograft mouse model of thyroid cancer, tumor growth was enhanced when co-injected with human mast cells due to an increase in tumor proliferation and vascularization [82]. Treatment with cromolyn significantly reduced tumor cell proliferation and growth. It should be noted that cromolyn did not impact tumor growth in this xenograft model in the absence of the human mast cells. In a study of MYC-induced pancreatic neuroendocrine tumors, MYC activation was associated with mast cell recruitment that was required for tumor growth, and treatment with cromolyn sodium prevented mast cell degranulation and decreased tumor growth [83]. More recently, in a gastric adenocarcinoma mouse model, it was demonstrated that gastric tumor cells produced IL-33, which resulted in mast cell activation that led to the production of macrophage-attracting factors CSF-2, CCL3, and IL-6 and subsequent tumor growth [58]. Cromolyn sulfate was successfully used in this model to decrease macrophage recruitment to the tumor microenvironment, tumor angiogenesis, tumor proliferation, and ultimately tumor growth. As described above with the 4T1 breast cancer tumor model, however, cromolyn is not universally effective as a monotherapy across tumor types, likely due to the context-dependent role of mast cells. While cromolyn is clinically effective in the treatment of allergic diseases and systemic mastocytosis, we do not have clinical trials at this time evaluating its efficacy in cancer patients.

#### 4.2.2. Targeting the FcεR1 Signaling Pathway

Rather than stabilize and prevent the release of mast cell mediators, upstream intracellular signaling pathways within mast cells can alternatively be targeted. IgE binding to the FcεRI results in FcεRI aggregation, then downstream phosphorylation of immunoreceptor tyrosine-based activated motifs on the receptor subunits, activation of spleen tyrosine kinase (SYK), phosphoinositide 3-kinase (PI3K), and Bruton’s tyrosine kinase (BTK) with ultimate downstream release of inflammatory mediators [84]. In allergic disorders such as asthma, anti-IgE monoclonal antibodies that inhibit the crosslinking of the FcεRI have been developed to prevent mast cell activation and degranulation. Omalizumab is an anti-IgE humanized monoclonal antibody that has been shown to be effective in severe allergic asthma and is commonly prescribed to patients today [85]. From the perspective that mast cell and mast-cell-induced inflammation is favorable in inducing anti-tumor responses, anti-tumor IgE antibodies have been proposed. Especially in tumors with high mast cell infiltration, the high density of FcεRI and longer half-life of antibodies compared to IgG antibodies make this an attractive therapeutic modality. Anti-tumor mast cell degranulation and decreased tumor cell growth were observed with tumor targeted humanized monoclonal anti-HER-2/neu IgE and also the humanized anti-CD20 IgE in in vitro studies [86]. Anti-MUC-1 IgE in an MUC-1-expressing 4T1 murine breast cancer model in combination with mast-cell-attracting chemokines led to tumor rejection, and importantly also led to the rejection of 4T1 cells subsequently on the contralateral flank in the absence of either the IgE antibody or chemokines, suggesting a memory immune response [87]. Of note, anti-tumor IgE antibodies are limited to targetable tumor antigens such as HER2, CD20, and MUC-1 as above.

Downstream of IgE binding to FcεRI, the signaling cascade can be inhibited by blocking early stimulatory signals such as SYK, PI3K, and BTK. These proteins are being targeted in allergic diseases in order to target mast cell mediators and are also either approved or in clinical testing in cancers, though not specifically for the purpose of targeting mast cells. For example, the PI3K alpha-specific inhibitor alpelisib has been studied in allergic rhinitis and is used in estrogen-receptor-positive metastatic breast cancer [88,89]. PI3K delta and PI3K gamma-specific inhibitors are undergoing clinical testing in multiple cancers, especially in the context of combination immunotherapy. It will be informative to the field of mast cell cancer therapy to study pharmacodynamic changes in intratumoral mast cells and mediators in patient samples treated with these therapies, and to study the mast-cell-specific role of these therapies in pre-clinical studies. Alternatively, there have been attempts at stimulating inhibitory signaling pathways in mast cells such as SHIP-1 (Src homology 2 domain-containing inositol 5′ phosphatase 1) phosphatase. The phosphatase SHIP-1 can inhibit the above signal by dephosphorylating the stimulatory product of PI3K activation. A SHIP-1 activator AQX-1125 is currently undergoing clinical testing in allergic asthma [90]. A limitation of targeting the SYK/PI3K/BTK/SHIP-1 pathway is that the enzymes have widespread expression across cell types, so toxicity is a significant concern in drug development.

#### 4.2.3. Stimulating Toll-Like Receptors to Modulate Mast Cells towards an Anti-Tumor Phenotype

Given mast cell abundance in many solid tumors and their plasticity that ranges from pro-tumorigenic to anti-tumorigenic function, there are also significant efforts to favorably manipulate already existent intra-tumoral mast cells towards an anti-tumor phenotype rather than deplete them. Targeting toll-like receptors (TLR) presents a viable therapeutic strategy to achieve this either directly with synthetic TLR agonists or indirectly via intermediate natural TLR agonists that are secreted in the body in response to other immunotherapies. TLRs are membrane pattern recognition receptors expressed on both immune and some non-immune cells that recognize structurally conserved molecules derived from microbes such as lipopolysaccharide (LPS), flagellin, and unmethylated CpG oligodeoxynucleotide DNA. In response to receptor activation, TLRs recruit adaptor proteins to initiate a downstream signaling cascade that results in an innate immune response with resultant antigen-specific adaptive immune responses. The field of TLR cancer immunotherapy has historically focused on enhancing TLR activity on dendritic cells, macrophages, and even B cells, but there is growing appreciation of the importance of TLRs on mast cells in shaping cancer immunity. Human mast cells have been shown to express the majority of TLRs—though the specific expression and role of the TLRs are likely context dependent—and are now recognized to mediate the effects of several cancer immunotherapies [91].

In the poorly immunogenic aggressive B16.F10 melanoma model, the TLR2 agonist, Pam_3_CSK_4_, was shown to inhibit tumor growth in a mast cell TLR2-dependent manner; tumor control was significantly reduced in the mast-cell-deficient Kit*^W-sh/W-sh^* murine background or in mice with TLR2-deficient bone-marrow-derived mast cells compared to wildtype mast-cell-containing mice [92]. This anti-tumor effect was mediated by recruitment of CD8 T cells and NK cells likely by CCL3 secretion, IL-6 secretion potentially leading to an anti-proliferative impact on tumor cells, and also reduced mature blood vessel density. The latter highlights that repolarizing mast cells promotes favorable anti-tumor effects but also decreases tumor-promoting properties such as vascular proliferation associated with the suppressive tumor-infiltrating mast cells. In another melanoma study with B16-F10 and M3 tumor models, the TLR7 agonist, imiquimod, was observed to significantly increase mast cell production of the chemokine CCL2 in a TLR7-dependent fashion, which subsequently leads to the recruitment of effector plasmacytoid dendritic cells that ultimately mediate cell killing and tumor control [93]. This is another example of mast cells being critical intermediates for the efficacy of TLR agonist cancer immunotherapy.

Mast cells have also recently been shown to facilitate responses to immune checkpoint inhibitor therapy. Kaesler et al. identified a systemic lipopolysaccharide signature in melanoma patients treated with anti-CTLA-4 immune checkpoint blockade that developed immune-mediated colitis and asked if this LPS activation at the tumor site may contribute to cancer treatment responses. Using an ovalbumin-expression B16 melanoma model, they observed a partial reduction in tumor volumes with adoptive transfer of tumor-specific T cells. However, when tumor-specific T cells were combined with a peritumoral LPS injection, complete tumor control was achieved. The authors then went back to patient tumors to hypothesize potential mediators of LPS activation and observed that mast cells were highly infiltrated in tumors with spontaneous immune regression. They subsequently tested the role of mast cells in their murine models by studying the effect of LPS exposure in two mast-cell-deficient models, which failed to mount an immune response in contrast to wildtype mice. Furthermore, reconstituting these mast-cell-deficient models with bone-marrow-derived mast cells restored the LPS-induced anti-tumor immune response. Notably, this was shown to be TLR4 dependent as well as downstream NF-κB dependent. This mast cell LPS-mediated TLR4 activation led to subsequent mast cell secretion of CXCL10, a chemokine that attracts effector T cells to tumors, which then mediate anti-tumor immunity [94].

#### 4.2.4. Targeting Inhibitory Receptors and Ligands

Antibodies targeting inhibitory cell surface receptors to inhibit mast cell activation are another avenue of active research. Recently, SIGLEC-8 was identified as an inhibitory receptor primarily present on the cell surface of mast cells, eosinophils and to a lesser degree basophils [95,96], and when engaged by its ligand leads to direct antibody-dependent cell-mediated cytotoxicity and decreased degranulation. Antolimab is a humanized IgG1 monoclonal antibody that agonizes SIGLEC-8 leading to decreased mast cell activation and decreased inflammation in mouse models of anaphylaxis. It is currently being studied in numerous clinical trials of allergic disorders and if safe and effective could be expanded to clinical studies of solid tumor patients. FcγRIIB and CD300a are other known inhibitory receptors on mast cells. FcγRIIB bi-specific antibodies and recombinant Fcγ and Fcε recombinant fusion proteins that recognize both FcεR1 and FcγRIIB have been shown to suppress human mast cells and prevent anaphylaxis in in vivo studies [97,98,99,100]. Similarly, bispecific antibodies linking CD300a to IgE-bound FcεR1 have been shown to downregulate mast cell activation and allergic processes in pre-clinical models [101]. With increasing awareness that PD-L1 expressing myeloid cells are a critical mediator of PD-1/PD-L1 immune checkpoint blockade, emerging data suggest that mast cells may play a role in this pathway. In a gastric cancer mouse study, tumor cell-derived TNF-α was shown to increase the number of intratumoral mast cells expressing the inhibitory ligand PD-L1 which resulted in the suppression of T-cell immunity [52]. Blockade of mast-cell-associated PD-L1 resulted in enhanced tumor control, CD3 T-cell infiltration, as well as increased IFN-γ and granzyme B production. As we identify which clinical settings are most appropriate for the inhibition of mast cell activation or inhibitory PD-L1, clinical trials in cancers with these agents can be appropriately designed.

### 4.3. Modulating Effects of Mast Cell Mediators

Directly modulating the effects of mast cell mediators after secretion is a final therapeutic approach to alter mast cell downstream activation, including targeting histamine or histamine receptors, proteases such as tryptase, and TNF-α. The effect of histamine is highly tumor type dependent and has been shown to be both tumorigenic and have anti-tumor effects depending on which histamine receptor (H1–H4) is activated and other local cues as discussed above, complicating clinical translation. Along these lines, preclinical and retrospective studies have supported both histamine receptor agonists or antagonists in different settings [102]. In patients, systemic administration of histamine combined with IL-2 was found to improve the efficacy of IL-2 therapy, potentially due to augmented NK cell mediated tumor cell cytotoxicity [103,104]. Histamine antagonism has been studied with mixed results. A pre-surgical trial of the H2 receptor antagonist cimetidine in breast cancer did not influence tumor cell proliferation, though changes in immune cells were not tested in this study [105]. Another trial of the H2 receptor antagonist famotidine in the preoperative setting in a breast cancer cohort showed an increase in tumor-infiltrating lymphocytes [106], but no clinical or anti-cancer endpoints were assessed. In colorectal cancer, a Cochrane systematic review of six randomized control trials [107,108,109,110,111,112] of H2 receptor antagonists demonstrated improved overall survival (HR 0.70) with pre- and/or postoperative therapy in patients who have had surgical resection with curative intent [113]. The modern relevance of these findings, however, is limited by more effective adjuvant chemotherapy being available for high-risk patients compared to the control arms used in these colorectal cancer trials.

Tryptase is another mast cell mediator released during mast cell activation and promotes angiogenesis and degradation of the extracellular matrix, resulting in cancer growth, cell invasion, and metastasis. Tranilast, nafamostat mesylate, and gabexate mesylate are three mast cell tryptase inhibitors that have been shown preclinically to have anti-cancer activity across multiple solid tumors either as monotherapy or in combination with other cancer therapies, with the majority of studies focused on pancreatic, colorectal, and breast cancer [114]. Uwagawa et al. studied the effect of nafamostat mesylate in combination with gemcitabine in a phase II clinical trial in 35 patients with unresectable locally advanced or metastatic pancreatic cancer and found the drug decreased circulating levels of tumor marker CA19.9, improved quality of life, and had a median overall survival of 10.0 months with a 40% 1-year survival rate [115,116]; this combination was not further pursued, as combination chemotherapy regimens became standard of care. In a recent study, tranilast synergized with liposomal doxorubicin and dual immune checkpoint inhibition (anti-CTLA4 and anti-PD1 inhibitors) in triple negative breast cancer mouse models, where it was found to restore perfusion and oxygenation and restore T-cell infiltration [117]. Of note, while tryptase inhibitors decrease angiogenesis and inhibit matrix metalloproteinases, they have additional effects, including the suppression of the immunosuppressive cytokine TGF-β, inhibition of additional proteases, NF-κB down regulation, and MAP kinase pathway inhibition [114].

TNF-α is another mast cell mediator that has long been implicated in the pathogenesis of inflammatory bowel diseases such as ulcerative colitis and Crohn’s disease where TNF-α inhibitors such as infliximab have been mainstays of therapy. In a study of colitis, treatment with infliximab led to a significant decrease in the development of colorectal cancers [118]. TNF-α has been shown to be detrimental for immune checkpoint blockade by upregulating the secondary checkpoint component TIM-3 on ICB-induced CD8 T cells [119]. The safety of the triple combination of ipilimumab, nivolumab and an antibody inhibiting TNF-α (infliximab or certolizumab) is being studied in a phase I clinical trial in advanced melanoma (NCT03293784). In cancer, TNF-α has had dual roles and in addition to promoting cancer through chronic inflammation, also has direct cytotoxic effects and stimulates anti-tumor immunity as discussed above. Given toxicity concerns, TNF-α has been deemed unsafe to administer systemically but has shown some efficacy in isolated limb perfusion in melanoma and sarcoma, where it has ≥80% objective response rates [90]. In colon cancer, direct injection of TNF-α into liver metastases has shown to be an effective therapy in preclinical testing and was shown to have acceptable safety in a small phase I trial of colon cancer patients [120].

Additional mediators such as the protease chymase, prostaglandins, leukotrienes, cytokines, chemokines, and growth factors may also be relevant targets, but, as with the other mediators, specificity to mast cells is limited.

## 5. Conclusions and Future Directions: Importance of Translational Research

Translational data from patient tumor tissues across a range of solid tumors and preclinical studies strongly indicate that mast cells are critical determinants of anti-tumor immunity and cancer outcomes. Existing work suggests that mast cells may be key orchestrators of the initial anti-tumor immune response but also a mechanism of resistance to immune checkpoint blockade as well as other cancer therapies. As mast-cell-targeting therapies are increasingly used and studied in allergic diseases, their application to cancer presents an exciting and practical frontier in cancer immunotherapy. Possible therapeutic avenues include c-KIT inhibitors, mast cell stabilizers, FcεR1 signaling pathway activators/inhibitors, antibodies targeting inhibitory receptors and ligands, TLR agonists, and modulators of mast cell mediators. Especially compelling are newer studies showing synergy and durable responses induced by combination with immune checkpoint blockade. However, clinical translation is limited by mast cell phenotypic plasticity and context dependence, with different anti-tumor and pro-tumor ramifications depending on specific biologic circumstances such as tumor type, mast cell location in the tumor microenvironment and the activation status of the mast cells.

For optimal translation of mast-cell-directed therapies to the clinic, it is imperative to incorporate patient tissue-based translational research to study the biologic relevance and therapeutic efficacy of mast-cell-directed therapies. First, our understanding of relevant mast cell biology is limited by a substantial portion of studies conducted in vitro with human and murine samples, inherent differences in mast cell biology between humans and experimental murine models, and difficulty in showing specificity of mast cell mediators such as cytokines and chemokines to mast cells as they can be secreted by multiple cell types. In addition, the context dependence of mast cells necessitates a comprehensive understanding of the relevant context not only of the background cancer—cancer type, stage, likely treatment history and concurrent anti-cancer therapies—but also of the mast cells’ activation status, location within tumor and how they are being altered by the investigational agents of interest. Advances in immune monitoring are allowing for the in-depth profiling of immune cells with single cell sequencing technologies, functional assays that enable the assessment of polyfunctional responses, and multiplexed immunohistochemistry that allow for an understanding of spatial organization and interaction between cells. Together, this type of research will not only be critical in advancing the field by identifying targetable features and pathways in mast cells that can be therapeutically manipulated but also how these change longitudinally with other systemic therapies to determine optimal therapeutic combinations. As preclinical investigations such as combination studies with PD-1 immune checkpoint blockade in melanoma suggest, it may be identifying optimal therapeutic combinations that will be most successful rather than mast cell targeting as monotherapy. Importantly, in designing the clinical trials to test such novel combinations, it will be important to pair rationale trial design with pharmacodynamic assessments to best identify mechanisms of treatment response and resistance. Clinical assessment of toxicities will also be critical to ensure that while attempting to favorably modulate the anti-tumor properties of mast cells, biological responses critical to maintaining the host’s state of health are not adversely perturbed. Together with continued preclinical mechanistic studies, such a translational research approach will allow us to most effectively extrapolate the success of mast-cell-directed therapies in other diseases to the field of cancer immunotherapy.

## Data Availability

Not applicable.
